# Editorial for the Special Issue on Interface Circuits for Microsensor Integrated Systems

**DOI:** 10.3390/mi9100527

**Published:** 2018-10-17

**Authors:** Giuseppe Ferri, Vincenzo Stornelli

**Affiliations:** Department of Industrial and Information Engineering and Economics, University of L’Aquila, 67100 L’Aquila, Italy

Recent advances in sensing technologies, especially those for Microsensor Integrated Systems, have led to several new commercial applications. Among these, low voltage and low power circuit architectures are a focus of growing interest, being suitable for portable long battery life devices. The aim is to improve the performances of actual interface circuits and systems, both in terms of voltage mode and current mode, in order to overcome the potential problems due to technology scaling and different technology integrations. Related problems, especially those concerning parasitics, lead to a strong interest in interface design; particularly, analog front-end and novel and smart architecture must be explored and tested, both at simulation and prototype level. Moreover, the growing demand for autonomous systems is more difficult to meet in the interface design due to the need for energy-aware cost-effective circuit interfaces integration and, where possible, energy harvesting solutions. The objective of this Special Issue has been to explore the potential solutions to overcome actual limitations in sensor interface circuits and systems, especially those for low voltage and low power Microsensor Integrated Systems. The present Special Issue presents and highlights the advances and the latest novel and emergent results on this topic, showing best practices, implementations, and applications.

There are 10 papers published in this Special Issue, covering micromachined sensors interfacing circuits [1,2,3,4], techniques for sensor interrogation and conditioning circuits [5,6,7], and sensors and systems design [8,9,10].

In particular, Malcovati et al. presented an overview of MEMS microphones evolution interfacing based on actual design examples, focusing on the latest cutting-edge solutions [1]. Kim et al. proposed a reconfigurable sensor analog front-end using low-noise chopper-stabilized delta-sigma capacitance-to-digital converter (CDC) for capacitive microsensors [2]. Qiao et al. addressed an alternative to capacitive MEMS accelerometers interface circuits, conventionally based on charge-based approaches, based on frequency-based readout techniques that have demonstrated they have some unique advantages [3]. Pantoli et al. proposed a novel interface circuits for micromachined silicon photomultipliers based on a second-generation voltage conveyor as an active element, performing as a transimpedance amplifier [4]. On the interrogation and conditioning circuits side, Hu et al., in order to match the high output impedance of Tribo-electric-Nano-generator (TENG) and increase the output power, presented an adaptable interface conditioning circuit, which is composed of an impedance matching circuit, a synchronous rectifier bridge, a control circuit, and an energy storage device [5]. D’Amico et al. presented the study of useful electrical properties of directly coupled L–C cells forming a discrete ladder network (L–C L.N.) to be applied to the sensor field up to be applied on a large scale down to micrometric dimensions in agreement with the technologic ability to shrink the capacitive sensor dimensions [6]. Demori et al. proposed an interrogation techniques and interface circuits for coil-coupled passive sensors: the interrogation of sensor units is based on resonance, denoted as resonant sensor units, in which the readout signals are the resonant frequency and, possibly, the quality factor [7]. On the sensors and systems design, Wei and Bao presented a low power, energy-efficient precision CMOS temperature sensor based on bipolar junction transistors and a pre-bias circuit and bipolar core [8]. Wu et al. presented an A variable-gain chopper-stabilized instrumentation amplifier (chopper IA), which employs a low pass filter (LPF) to attenuate the up-converted noise at the chopping frequency for micromachined sensors applications [9]. Liu et al. presented a review of recent progress in the rapid sintering of nanosilver pastes: preparation of nanosilver particles and pastes, mechanisms of nanopastes sintering, and different rapid sintering processes were discussed [10]. 

The guest Editors would like to take this opportunity to thank all the authors for submitting their papers to this special issue and also want to thank all the reviewers for dedicating their time and helping to improve the quality of the submitted papers. 

## References

[1] Malcovati P., Baschirotto A. (2018). The Evolution of Integrated Interfaces for MEMS Microphones. Micromachines.

[2] Kim H., Lee B., Mun Y., Kim J., Han K., Roh Y., Song D., Huh S., Ko H. (2018). Reconfigurable Sensor Analog Front-End Using Low-Noise Chopper-Stabilized Delta-Sigma Capacitance-to-Digital Converter. Micromachines.

[3] Qiao Z., Boom B., Annema A., Wiegerink R., Nauta B. (2018). On Frequency-Based Interface Circuits for Capacitive MEMS Accelerometers. Micromachines.

[4] Pantoli L., Barile G., Leoni A., Muttillo M., Stornelli V. (2018). A Novel Electronic Interface for Micromachined Si-Based Photomultipliers. Micromachines.

[5] Hu Y., Yue Q., Lu S., Yang D., Shi S., Zhang X., Yu H. (2018). An Adaptable Interface Conditioning Circuit Based on Triboelectric Nanogenerators for Self-Powered Sensors. Micromachines.

[6] D’Amico A., Santonico M., Pennazza G., Zompanti A., Scipioni E., Ferri G., Stornelli V., Salmeri M., Lojacono R. (2018). Resonant Directly Coupled Inductors–Capacitors Ladder Network Shows a New, Interesting Property Useful for Application in the Sensor Field, Down to Micrometric Dimensions. Micromachines.

[7] Demori M., Baù M., Ferrari M., Ferrari V. (2018). Interrogation Techniques and Interface Circuits for Coil-Coupled Passive Sensors. Micromachines.

[8] Wei R., Bao X. (2018). A Low Power Energy-Efficient Precision CMOS Temperature Sensor. Micromachines.

[9] Wu C., Chen H., Yen M., Yang S. (2018). Chopper-Stabilized Instrumentation Amplifier with Automatic Frequency Tuning Loop. Micromachines.

[10] Liu W., An R., Wang C., Zheng Z., Tian Y., Xu R., Wang Z. (2018). Recent Progress in Rapid Sintering of Nanosilver for Electronics Applications. Micromachines.

